# Narrative Review: Nutrient Deficiencies in Adults and Children with Treated and Untreated Celiac Disease

**DOI:** 10.3390/nu12020500

**Published:** 2020-02-15

**Authors:** Johanna M. Kreutz, Marlou P. M. Adriaanse, Elisabeth M. C. van der Ploeg, Anita C. E. Vreugdenhil

**Affiliations:** 1 Department of Paediatrics and NUTRIM School of Nutrition and Translational Research in Metabolism, Maastricht University Medical Centre, P. Debyelaan 25, 6229 HX Maastricht, The Netherlands; johanna.kreutz@mumc.nl (J.M.K.); marlouadriaanse@hotmail.com (M.P.M.A.); 2 Department of Dietetics, Maastricht University Medical Centre, P. Debyelaan 25, 6229 HX Maastricht, The Netherlands liesbeth.vander.ploeg@mumc.nl

**Keywords:** celiac disease, gluten free diet, nutrient deficiencies, nutritional status, supplementation

## Abstract

Nutrient deficiencies are well recognized as secondary consequences of celiac disease (CD) and closely related to the clinical presentation of affected patients. Despite their clinical significance, consensus is lacking on the pattern and frequency of nutrient deficiencies in CD, the usefulness of their assessment at the time of diagnosis and during follow-up. This review aims to provide an overview of nutrient deficiencies among pediatric and adult CD patients at diagnosis and on a gluten-free diet (GFD), and their potential causes in CD. Secondly, we review their impact on CD management strategies including the potential of nutrient supplementation. A search of Medline, Pubmed and Embase until January 2019 was performed. Despite a high variability between the reported deficiencies, we noted that nutrient deficiencies occur frequently in children and adults with CD at diagnosis and during treatment with a GFD. Both inadequate dietary intake and/or diminished uptake due to intestinal dysfunction contribute to nutrient deficiencies. Most deficiencies can be restored with (long-term) treatment with a GFD and/or supplementation. However, some of them persist while others may become even more prominent during GFD. Our results indicate a lack of comprehensive evidence on the clinical efficacy of nutrient supplementation in CD management highlighting the need for further studies.

## 1. Introduction

Celiac disease (CD) is a common immune-enteropathy triggered by dietary gluten in genetically susceptible individuals [1]. In CD, the immunologic response to gluten peptides causes histological abnormalities in the small intestine. These histological aberrations such as villous atrophy reduce the functional capacity of the intestine [1]. A clinically relevant consequence is malabsorption resulting in an increased risk for nutritional deficiencies. These deficiencies can contribute to clinically important comorbidities such as anemia, osteoporosis and depression [2,3,4]. Nutritional deficiencies do not only play a role at the time of diagnosis, but also during treatment with a gluten-free diet (GFD).

The functional absorptive surface of the intestine in CD is expected to restore after treatment with a GFD, thereby reestablishing nutrient absorption. However, studies reveal that full histological recovery requires long-term treatment, especially in adult patients. This makes CD patients prone to nutrient deficiencies in the first period after initiation of a GFD, even when strictly adhering to the diet [5,6,7,8]. Another complicating factor that may trigger development of deficiencies while treating the disease with a GFD is the diet itself. Withdrawing gluten-containing foods from the diet and substituting them with cereals that are less rich in nutrients may lead to a nutritional imbalance contributing to an overall impaired nutrient status [9,10,11]. Currently, it is unclear which types of nutrient deficiencies are frequently present in CD patients at diagnosis and during treatment with a GFD. Moreover, there is a lack of knowledge on the clinical relevance of nutrient deficiencies and hence the potential impact of nutrient supplementation on the clinical outcome in CD. To answer the main research question that forms the basis of this narrative review, we reviewed the published scientific evidence for nutrient deficiencies in pediatric and adult CD patients at diagnosis and during treatment with a GFD. Moreover, the review provides an appraisal of underlying causes of deficiencies and the clinical relevance of nutrient supplementation. Currently, the clinical impact of nutrient deficiencies and the effectiveness of their treatment are currently poorly defined. Besides, the assessment of nutrient deficiencies can be executed by different approaches, including measuring the values of individual nutrients in blood or urine, or by indirect evaluation of putative clinical consequences of deficiencies. Both of the latter approaches are addressed in this review. Finally, the review aims to evaluate whether the currently available evidence is of sufficient quality to provide recommendations for clinical practice.

## 2. Materials and Methods 

For this narrative literature review, as defined by Grant et al., a search of Medline, Pubmed and Embase from 1960 until January 2019 was performed to identify potentially relevant publications [12]. The following search terms were used to search in titles and abstracts using “All fields”, as well as MeSH terms when available: ‘celiac disease’, ‘coeliac disease’, ‘nutrient status’, ‘dietary intake’, ‘vitamins’, ‘minerals’, ‘nutrients’, ‘gluten-free diet’, ‘histology’, and ‘histological recovery’. Additionally, a search with relevant nutrients was conducted including the search terms ‘iron’, ‘ferritin’, ‘vitamin D’, ‘vitamin B6’, ‘vitamin B12’, ‘calcium’, ‘folic acid’, ‘zinc’, ‘magnesium’. The search was limited to studies in the English language and full-text availability. This approach resulted in a total of 8478 articles for potential inclusion in this review. The search was not narrowed down further, to make sure as many eligible articles as possible were included in this overview. All potentially relevant original articles were screened for inclusion by two researchers in a step-wise approach; first, based on title, then on screening of the abstracts, and then by full-text screening of the remaining articles. Reference lists from the selected articles were also screened manually for relevant publications. The cited articles were selected based on the relevancy to the review objectives. Included were original studies on histological recovery in celiac patients on a GFD; nutrient intake of celiac patients; and nutrient deficiencies in untreated or treated celiac patients. As there is currently no clear consensus on the definition of nutrient deficiencies and how these should be evaluated, studies were included that measured serum nutrient values. Furthermore literature describing the link between nutrient levels and associated symptoms and comorbidities in CD were added to provide a general overview. Articles describing nutrient deficiencies were only included when they reported either the percentage or total number of patients with a nutrient deficiency. An appraisal of the quality of the studies was conducted to select articles of high quality which were summarized in the results of this review. The tables report the percentages of patients or of a reference population with nutrient levels below reference point and nutrient intake below recommendation (Table 1 and Table 2 respectively). For both the reference values for nutrient concentrations and reference values for recommended nutrient intake, the values chosen by the individual studies were used, meaning that the cut-off values vary between studies. Furthermore, this entails that no distinction was made in the severity of the nutritional deficit. This means, that the reported percentage of patients with a deficiency include those with severe deficiencies as well as sub-optimal values below the cut-off value. No selection was made based on the technique chosen to measure nutrient values and an overview of the selected methods was included when reported by the authors (see Supplementary Table S1).

Only recent studies, not older than 15 years, were included in the tables. Furthermore, the study population had to be well defined with sound confirmation of CD diagnosis, as well as the moment of assessment, specifically differentiating between the moment of CD diagnosis and assessment of follow-up. Only those articles were selected that presented the results in a way that they could be extracted for this review. This included a clear differentiation between groups and definition of the moment of measurements. Furthermore, the results had to provide either percentages or numbers of subjects with nutrient deficiencies or insufficient intake of certain nutrients. Consequentially, articles only providing mean or median values of nutrient levels or nutrient intake within the groups were excluded. Single-case reports were not included. Level of evidence was assessed according to the 2009 Oxford Centre for Evidence-Based Medicine (OCEBM) [13,14]. The evidence level could be graded down based on the researchers’ assessment of study quality and relevance and could be graded up in case of a large or very large effect size. An overview of all studies included in Table 1 and Table 2 is provided in the Supplementary Material of this review. The overview includes subject characteristics and demographics of each study, as well as outcome parameters and details on the information provided by the authors concerning chosen diagnostic tests chosen and cut-off values for nutrient assessment. Lastly, the level of evidence was provided according to the 2009 OCEBM (see Supplementary Table S1). Due to the small number of recent, high-quality publications on several nutrient values that met all the aforementioned criteria, other studies that did not meet these criteria were included in the review as well. These studies were included as the best available evidence and were highlighted within the text and tables as being of lower quality.

## 3. Nutritional Deficiencies in Celiac Disease at Diagnosis and on a Gluten-Free Diet

An overview of the most important nutrient and mineral deficiencies reported in CD at diagnosis and on a GFD are summarized in Table 1. Percentages of nutrient deficiency in healthy reference populations are mentioned in Table 1 as well. The majority (69%) of the studies summarized in this review were conducted in Europe, 13% in North America, and 9% in India, the remaining studies being conducted in Israel, Australia and South America. Half of the studies were conducted prospectively, while the other half were mainly retrospective chart reviews or cross-sectional studies (see Supplementary Table S1). 

### 3.1. Nutritional Deficiencies at Moment of Diagnosis in Untreated Celiac Disease

Twenty-nine studies that evaluated nutritional deficiencies in CD at diagnosis were identified, only 15 of which describe nutritional status in children. The most frequently described deficiencies in CD patients at diagnosis are of iron, vitamin D, calcium, vitamin B12, folic acid and zinc. Presence of iron deficiency was described in 6%–82% of adult patients and in 12%–82% of pediatric patients newly diagnosed with CD [15,16,17,18,19,20,21,22,23,37,38,39]. Iron status has been well researched in regard to CD and the results presented here were obtained from twelve different study populations. 

Vitamin B12 (cobalamin) deficiency has been reported in 5%–19% of untreated CD patients [16,17,18,20,22,23,32,37]. Folic acid deficiency (vitamin B9) has been described in 11% up to, as many as 75% of adults with untreated CD and 14%–31% of children [15,16,17,18,19,20,22,23,31,32,37,38].

The prevalence of deficiencies in several micronutrients in CD is unclear. This is due to lack of scientific evidence or high variety between studies. This is the case in vitamin B6 deficiency, for instance, which has recently only been studied in two adult but no pediatric CD cohorts. A recent study from Wierdsma et al. showed that vitamin B6 deficiency was present in 15% of untreated adult CD patients but not in controls [18]. In contrast, other studies reported similar vitamin B6 levels in adult CD patients compared to healthy controls [49].

Vitamin D levels were demonstrated to be low in 5%–88% of untreated adult patients, and in 0%–70% of untreated pediatric patients [18,25,26,27,40,41,42,45]. Circulating levels of calcium were described to be low in 0%–26% of untreated adults and 0%–41% of pediatric patients [17,19,37,40,45,46]. The only prospective case-control study of good quality investigating hypocalcemia was conducted by Zanchi et al. which found a high prevalence of 40.7% of hypocalcemia at diagnosis in pediatric CD patients, compared to 0% in controls [40]. Similarly, the evidence of higher quality available for vitamin D levels also indicates a high prevalence of vitamin D deficiency in pediatric CD patients. Tokgöz et al. found a prevalence of vitamin D deficiency in 92.4% of CD patients compared to 18% in controls. This difference becomes even more profound when separately investigating vitamin D insufficiency (levels below 30 ng/mL) which was present in 61.5% of CD patients and merely 4% of healthy controls [42]. 

Next to calcium, deficiencies in other minerals and elements are associated with CD, although literature reporting on these is particularly scarce. Magnesium deficiency was reported in 7%–11% of untreated pediatric CD patients [40,48]. Magnesium deficiency in untreated adult CD patients has only been reported in two studies that did not meet the criteria for the quality assessment, being published longer than 20 years ago, indicating a prevalence of magnesium deficiency in 13%–17% [35,36]. Zinc deficiency has been found in 67% of untreated adult patients. Notably, all three studies reporting zinc levels in pediatric patients with active disease have found levels below the reference value in more than half of the patients, and a prospective randomized controlled trial conducted by Rawal et al. even found zinc deficiency to be present in more than 70% of patients [37,47,50]. 

Taken together, nutrient deficiencies are highly prevalent at time of CD diagnosis in the pediatric and adult population, although there is a variance in reported fractions of nutrient deficiencies. Importantly, not only routinely measured nutrients such as iron and vitamin B12 are deficient, but also less recognized and studied nutrients such as zinc appear to be deficient frequently in patients with active CD.

### 3.2. Nutritional Deficiencies While on a Gluten-Free Diet in Treated Celiac Disease

After diagnosis and institution of a GFD, restoring the nutrient status and maintaining adequate nutrient intake in CD patients remains a challenge. Studies reported that mean micronutrient levels increase in adult patients within one year of gluten elimination [17,23,32]. However, the velocity of this increase can vary, and an increase in nutrient concentrations does not always lead to full normalization. The rate of normalization as well as the time period necessary for nutrient levels to restore is of clinical importance, as it can impact health and disease outcomes in the short and long term. The selected studies mainly chose a time period of 6 to 12 months after institution of the GFD as indicators for short-term follow-up. Based on these results, few conclusions can be drawn on the time period necessary for recovery of nutrient deficiencies after diagnosis (see Table 1). When studying the prevalence of nutritional deficiencies in CD after diagnosis, these can be grouped into three categories: nutrient deficiencies that generally fully normalize during treatment; deficiencies that are more prevalent during follow-up than at the moment of diagnosis, and lastly, nutrients of which levels generally improve, yet not to the extent seen in a healthy reference population.

Nutrients that appear to be generally corrected in CD patients on a GFD are vitamin B12, folic acid, calcium and magnesium (see Table 1). Vitamin B12 deficiency has been described in 0% of treated adult CD patients by Vilppula et al. [17]. 

A GFD seems to also improve or even normalize folic acid levels in CD patients [17,23,24,30,32,51,52]. Folic acid deficiency in adults with CD on a GFD has not been reported in a study that met the quality assessment of this review, but it has been described in studies with a moderate quality. Hallert et al. reported low serum folate levels in 20% of adult CD patients on a 10-year GFD with a normalized intestinal mucosa in a study that met all criteria of our quality assessment except for the year of publication which was more than 15 years ago [33]. The prevalence of folic acid deficiency in treated children with CD has been studied in one recent publication that met our quality assessment. Wessels et al. describe a low prevalence of 0%–3% in their retrospective pediatric cohort on a GFD [38].

Likewise, resolution of calcium deficiency and to a lesser degree vitamin D levels was observed after institution of a GFD in most cases, which was confirmed in the study by Zanchi et al. with a high level of evidence [17,29,30,38,39,40,41,53]. Calcium levels have been described to be normal in all treated children with CD, while vitamin D was low in 0%–57% of children and in 0%–50% of adult patients on a GFD [29,30,38,39,40,41,53]. Next to the quality of the studies, it is important to consider that vitamin D levels especially are also low in the general population, when interpreting these results [25,40]. Additionally, the studies report nutrient deficiencies in the serum and do not take distribution and storage in the body into account. In the case of calcium and vitamin D for example, bone mineral density (BMD) and dental health are also a measure for total calcium in the body. Comorbidities associated with CD-related nutrient deficiencies are reviewed in the next section of this review. 

The effect of a GFD on magnesium levels is scarcely studied, yet appears to result in normalization of magnesium levels. Rujner et al. showed that the frequency of magnesium deficiency in treated adult CD patients (median 11 years on a GFD) was similar to that in untreated patients and controls [48]. In children, magnesium deficiency has been described in 4% of celiac patients on a GFD for 11 years [48].

In contrast to the aforementioned nutrient deficiencies that generally restore, other deficiencies might be even more prevalent on a GFD compared to the moment of diagnosis. Vitamin B6 deficiency might be an example of this. Studies reporting vitamin B6 levels in CD are, however, scarce and no study was available that met the inclusion criterion of this review which only included studies not older than 15 years. In a Swedish patient cohort of moderate quality published in 2002, low vitamin B6 plasma levels were found in 37% of adult CD patients on a long-term GFD, despite a recovered intestinal mucosa and dietary intake meeting recommendations [33]. 

Lastly, a considerable group of micronutrient imbalances improve, yet remain a prevalent problem on a GFD, iron deficiency being a chief example. Iron deficiency was described in 14%–41% of adult celiac patients on a GFD [54]. Strikingly, little evidence is available on the prevalence of iron deficiency in pediatric patients on a GFD and even less on the potential clinical implications in this regard. This should be given more attention, considering that iron deficiency anemia (IDA) is one of the most prevalent extra-intestinal manifestations of CD at diagnosis [21,38,55]. Adherence to a GFD does appear to reduce the prevalence and severity of IDA and increases serum iron and ferritin, although repletion of iron stores may take a prolonged time after healing of the intestinal mucosa [17,24,43,51,56,57]. In adult CD patients, the degree of histological recovery was associated with an increase in serum hemoglobin concentrations and impaired hemoglobin levels were evident in patients with incomplete mucosal recovery [57]. In pediatric CD patients on a GFD, the severity of villous atrophy correlated inversely with serum ferritin level, although no correlation with other hematological parameters was found [58]. Popov et al. showed a significant increase in mean serum ferritin concentration in pediatric CD patients after GFD in a retrospective chart review [59]. These findings were independent of the use of iron supplementation. Likewise, Wessels et al. showed a decrease in the proportion of pediatric CD patients with an iron deficiency after institution of a GFD [38,59]. In summary, iron deficiency appears to restore in a substantial proportion of CD patients after institution of the GFD. However, iron deficiency is still present in a considerable fraction of CD patients on a GFD.

In a similar way to iron deficiency, zinc deficiency appears to not improve sufficiently during CD treatment. A GFD has been shown to reduce zinc deficiency from a proportion of up to 72% of untreated CD patients to 16%–30% of patients on a GFD presenting with this deficiency [18,24,34,37,39,47,60]. Although zinc appears to restore in most patients, zinc deficiency was still found in 20%–30% of adult CD patients on a GFD [24,34]. 

When looking at hyperhomocysteinemia, controversial results have been found, and it is unclear whether this is generally corrected or remains a relevant consequence of CD even on a GFD. Studies on the occurrence of hyperhomocysteinemia showed a decrease of homocysteine levels after starting a GFD [32,49]. Dickey et al. reported normal levels in CD patients with recovered villous atrophy, while patients with persistent villous atrophy tended to have mildly elevated homocysteine levels [49]. However, Hallert et al. showed raised homocysteine levels in 17% of adult CD patients on a strict GFD for several years [33,61]. Also, levels of serum folate, vitamin B12 and vitamin B6 correlated inversely with homocysteine levels in CD patients [32,33,49,61]. Yet, Hadithi et al. reported that the presence and severity of CD were determinants of homocysteine levels, independent of measured B vitamin status [62].

### 3.3. Comorbidities Potentially Related to Nutrient Deficiencies in Celiac Disease 

**Figure 1:**
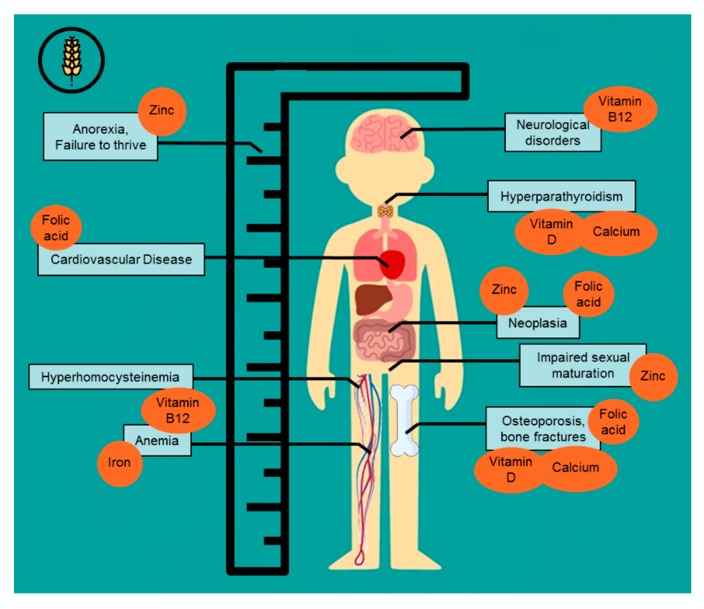
Comorbidities associated with nutrient deficiencies often found in celiac disease.

Nutrient deficiencies in CD can be the cause or a contributing factor to several symptoms and comorbidities associated with CD (see Figure 1). These, symptoms and comorbidities can serve as indicators for nutrient deficiencies in the long and short term. 

IDA is one of the most prevalent extra-intestinal manifestations of CD at diagnosis [21,55]. IDA can cause symptoms associated with CD such as fatigue, headaches and decreased exercise tolerance. Anemia in CD has further shown to be associated with greater disease severity and slower histological recovery in response to the GFD [63,64,65]. 

Other plausible causes of anemia in CD include vitamin B12 and/or folate deficiency, two nutrients essential in DNA synthesis. Deficiencies in these nutrients can limit DNA synthesis and result in megaloblastic anemia [66,67]. Vitamin B6 is important in hemoglobin formation, hence low levels of vitamin B6 can also contribute to the development of anemia in CD [68]. Therefore, anemia and its resulting symptoms can be a useful indicator for the importance of nutrient deficiencies in CD. 

Both vitamin B12 and folate, as well as vitamin B6 are also essential in the conversion of the harmful homocysteine. B vitamin deficiencies can therefore cause hyperhomocysteinemia in CD [66,67,69,70,71]. Consequentially, increased severity of celiac lesions is associated with higher homocysteine levels [62]. Aberrant homocysteine levels are related to an increased risk for venous thromboembolism, vascular disease, osteoporosis and adverse pregnancy outcomes [72,73,74]. 

Vitamin B12 deficiency has further been held responsible for several neurological symptoms which have been described in CD [66]. Folic acid deficiency has also been linked to a wide range of neurological disorders and conditions as diverse as neoplasia, cardiovascular disease and osteoporosis [67]. 

Other complications related to CD involve bone health, which is closely related to the nutritional status especially of calcium and vitamin D [75]. Impaired bone health is highly prevalent in children and adults at diagnosis of CD, even in individuals with mild enteropathy (without villous atrophy) [43,44,76,77,78,79,80,81,82,83,84,85]. In adult onset CD, a GFD generally improves but rarely normalizes BMD and the serious impact of this is shown in studies examining fracture risk [51,86]. Jafri et al. showed that fracture risk is increased in CD at diagnosis and during long term follow-up and a meta-analysis by Heikkilä et al. confirmed this increased risk for fractures in patients with CD [87,88].

In pediatric CD, the evidence on recovery of bone health and its impact on growth and long-term complications is more divergent. A strict GFD promotes an increase in BMD that may lead to complete recovery of bone mineralization within 1–2 years [40,43,44,45,77,78,79,80,81,82]. For instance, Mora et al. found that BMD and bone area normalized within a year of treatment in pediatric CD patients [78,79]. However, Kalayci et al. found that one year of strict GFD was insufficient for osteopenia to resolve in a substantial number of pediatric patients and suggests that BMD should be monitored in these children [78]. The influence that this could have on bone health in later life is uncertain. Moreover, the long term consequences of an initial impaired bone health and low calcium and vitamin D levels during childhood, regardless of its restoration, are not clear. 

Two other important minerals that can cause complications in CD patients when deficient, are zinc and magnesium. As summarized earlier, zinc deficiency is highly prevalent at diagnosis in pediatric and adult CD patient and seems to restore insufficiently when on a GFD [18,24,34,35,36,37,43,47,50,89]. 

Zinc is an essential trace element involved in numerous enzymatic reactions, biochemical functions and immune responses, and is required for growth and cellular function [90,91,92]. Zinc deficiency can alter protein synthesis, and in the absence of zinc growth retardation and impairment of sexual maturation occurs, which makes it particularly important for the pediatric CD population [92,93]. Classical presenting symptoms of CD such as anorexia and failure to thrive have therefore been linked to zinc deficiency [94]. Altuntas et al., found that zinc deficiency was present in more than half of the patients presenting with short stature that were diagnosed with CD in a pediatric population [50]. And although zinc deficiency appears to remain a relevant problem after institution of a GFD, little is known about the consequences of transient or persistent zinc deficiency in CD [47,89,94]. 

### 3.4. Role of Nutrient Supplementation in Celiac Disease Management

Overall, only few studies have systematically assessed the impact of nutrient supplementation as an additional therapy next to a GFD in CD. Some studies have suggested that nutrient supplementation may positively influence recovery, whereas others describe no difference between patients receiving supplementation and patients who do not. 

Foods rich in iron can be recommended in patients on a GFD, and supplements may be prescribed in clinical practice [9,10,11]. However, two prospective studies published in 1996 and 2003 found that even after iron supplementation for up to one year in pediatric CD patients, a significant number continued to have iron deficiency [43,58]. Additionally, a retrospective chart review showed that improvement of iron status in pediatric CD patients on a GFD was unrelated to the intake of supplementation [59]. It must, however, be noted that the use of supplementation was based on parent-reported medication history. 

In a study of vitamin status in adult CD patients after long-term treatment, circulating vitamin B12 as well as B6 levels were poorly correlated with dietary intake [33]. By contrast, vitamin B supplementation (folic acid, cyanocobalamin and pyridoxine) in CD patients on a long-term GFD has been shown to be effective in increasing serum vitamin B12 and B6 levels as well as serum folic acid levels [61,62]. Median serum levels were significantly higher in CD patients on B vitamin supplementation compared to both CD patients not using supplementation and healthy controls [62]. However, the role of each individual vitamin has not been established. 

Supplementation of vitamin B12 in patients on a GFD may be effective to prevent neurological complications associated with CD [95]. An increase in serum folic acid due to supplementation has been shown to be clinically relevant, since 6 months of supplementation improved anxiety and depressed mood in patients with longstanding treated CD [61].

Complications of poor bone health have far-reaching effects on a lifelong scale. Therefore additional measures improving bone health or at least preventing further bone loss in CD patients on a GFD might be beneficial. Calcium and vitamin D supplementation is considered a possible treatment option. However, neither vitamin D supplementation alone nor combined calcium and vitamin D supplementation for one year in adult CD patients provided additional benefit to the GFD with respect to BMD [96,97]. In children and adolescents with CD on a GFD, two years of combined calcium and vitamin D supplementation was reported to increase BMD, but levels did not normalize to those measured in healthy controls [98].

Supplementation in a methodologically sound randomized controlled trial of zinc for four weeks in newly diagnosed pediatric CD patients was not effective in increasing zinc levels compared to children who started a GFD without supplementation [47]. To our knowledge, the effect of supplementation of magnesium in CD patients has not been studied.

## 4. Causes of Nutritional Deficiencies in Celiac Disease

As shown above, nutritional imbalances are present in a substantial number of patients diagnosed with CD [99,100,101]. Nutrient deficiencies result from an imbalance between nutrient supply and biological need. In CD, nutrient supply can be insufficient due to impaired uptake on the one hand, and as a consequence of inadequate nutrient intake when on a GFD on the other hand. Impaired uptake is the main factor of nutrient deficiencies at diagnosis and becomes less important after histological recovery. After institution of a GFD, recovery of the small intestine occurs in most CD patients, making insufficient nutrient intake the factor most likely to contributing to nutrient imbalances, due to possible nutritional inadequacy of the GFD itself (see Table 2) [102,103,104,105,106].

### 4.1. Impaired Absorption DUE to Compromised Intestinal Epithelial Function

Absorption of macro- and micronutrients occurs in the small intestine, the site specifically affected in CD. Therefore, nutritional deficiencies due to diminished absorptive capacity are expected in active CD. Villous architecture and absorptive cells (enterocytes) are severely damaged and reduced in number in CD patients. Additionally, many enzymes necessary for digestion and absorption of nutrients are depleted or dysfunctioning, particularly brush border enzymes like the disaccharidases maltase, isomaltase, and lactase [114]. The disease affects the proximal small intestine and extends distally for a variable length in a more or less continuous fashion, with more severe damage in the proximal than in the distal part [54]. Consequently, mucosal impairment in CD at diagnosis may result in malabsorption of macro- and micronutrients, and studies show that more pronounced mucosal damage leads to increasing nutritional deficiencies [19,115,116]. 

The proximal small intestine is the site of absorption for iron, folate, and calcium [117]. Minerals and elements including zinc and magnesium are also mainly absorbed in the proximal small intestine, explaining why their levels may be low in CD patients [34]. Deficiencies in these nutrients can be explained by reduced absorptive capacity as well as reduced enzyme function in the affected proximal small intestine. 

Iron deficiency in untreated celiac patients is primarily caused by malabsorption due to loss of duodenal enterocytes. This results in a reduction of absorptive surface, as well as decreased amounts of the brush border enzyme ferrireductase, necessary for iron transport across the cellular membrane [117,118]. Moreover, enterocytes have an iron-storage capacity themselves which is directly affected by villous atrophy [117,118]. Although it has been previously suggested that gastrointestinal blood loss might also contribute to iron deficiency, recent studies suggest gastrointestinal bleeding to be uncommon in CD [119,120]. 

Diminished enzyme count and function also play a role in folic acid deficiency in untreated CD, since dietary folate, in the form of polyglutamates, is absorbed and cleaved to the monoglutamate form in the duodenum and jejunum, which are affected in CD [121]. 

Another example highlighting the complex causal cascade involved in development of nutrient deficiencies in CD is calcium. Potential factors involved are: loss of villous surface area, binding of calcium to unabsorbed fatty acids in the intestinal lumen, impairment of the active intestinal calcium transport mechanism due to depletion of vitamin D in enterocytes, and decreased dietary calcium and vitamin D intake secondary to concomitant lactase deficiency [122,123,124]. This shows that, for example, vitamin D deficiency can in turn enhance or cause other deficiencies, for example of calcium and magnesium. The small intestine normally absorbs up to 50% of magnesium intake, in part under the influence of active vitamin D [125,126]. 

More distal parts of the small intestine, jejunum and proximal ileum are responsible for absorption of carbohydrates, fat, vitamin B6 and fat-soluble vitamins (A, D, E, and K), while vitamins B12 absorption occurs in the terminal ileum [127]. Given the relative absence of villous atrophy in the (terminal) ileum, the mechanisms responsible for vitamin B12 deficiency in CD are unclear. In the ileum, vitamin B12 is absorbed bound to intrinsic factor, while a small portion is absorbed via passive diffusion along the entire small intestine. Hypothesized causes for vitamin B12 deficiency in CD include decreased secretion of gastric acid, dysfunctioning intrinsic factor, autoimmune gastritis, bacterial overgrowth or decreased efficiency of mixing with transfer factors [66,95,128].

### 4.2. Histological Recovery on a Gluten-Free Diet

Strict adherence to a GFD has classically been assumed to result in complete recovery of the intestinal mucosa and its absorptive function [129]. Indeed, elimination of gluten from the diet improves villous architecture and reduces the number of intraepithelial lymphocytes (IEL) [129]. However, despite its simple appearance, it is difficult to adhere to a strict GFD in the Western world where wheat is widely present in food (see Section 4.3). Inadvertent gluten intake may contribute to delayed mucosal recovery, as well as contamination of gluten-free products. Nevertheless, studies have shown that complete histological normalization is not always achieved in adult patients even when they maintain a strict GFD [5,7,129,130,131,132]. Currently, there is no full agreement on this topic. Therefore, insufficient histological recovery in CD is a potential reason for inadequate restoration or development of nutrient deficiencies even on a GFD and the mechanisms described above remain relevant in explaining causes of deficiencies in treated CD (see Section 4.1). In contrast, full histological recovery is almost always found in pediatric CD patients with good compliance to the GFD [8,133]. Moreover, the progress of histological recovery seems faster in children than adult CD patients [8,133]. 

Therefore, reduced absorptive capacity appears to be a less important cause of nutrient deficiencies in pediatric CD patients on a GFD [134]. Several studies performed to evaluate the histological recovery in adult CD patients after initiation of a GFD report a minority of CD patients achieving complete recovery of the intestinal mucosa within the first years of treatment. Long-term studies suggest that villous atrophy persists in 4%–79% of adult CD patients despite gluten elimination for an average of 8 years [5,7,129,130,131]. 

The wide range in reported histological recovery rates may be caused by the lack of prospective studies (selection bias), mixed adult and pediatric populations, various degrees of dietary strictness, a variable duration of gluten elimination prior to follow-up biopsies, and different definitions of mucosal and villous recovery. Nevertheless, authors agree that recovery of the celiac mucosa requires long-term treatment, especially in adult patients [6,7,129,130]. 

The slow and incomplete intestinal recovery in CD patients on a GFD might contribute to nutrient deficiencies. For instance, the degree of histological damage in CD patients has been shown to correlate with the severity of iron deficiency at time of diagnosis [18,19,24,27]. In addition, folic acid levels are generally depressed in patients with severe villous atrophy compared to patients with milder lesions. Although most studies reported no significant differences in vitamin B12 concentrations between patients with partial or (sub)total villous atrophy, others showed that vitamin B12 levels tended to correlate with the severity of the intestinal lesions [23,24,27,95,116,128]. However, no correlation has been found to date between the severity of the small intestinal lesion and vitamin D, B6, calcium, magnesium and zinc levels in celiac patients.

### 4.3. Nutrient Imbalance associated with a Gluten-Free Diet

Historically, the treatment of CD has focused on the avoidance of gluten-containing food and less importance has been devoted to the nutritional quality of the GFD [99]. However, maintaining a nutritionally adequate diet on a GFD requires effort and attention and insufficient dietary intake of nutrients is an important contributing factor to nutritional deficiencies in CD. The grains and gluten-containing products excluded in a GFD, are a major source of iron, dietary fiber, B vitamins and iodine, and contribute substantially to the energy and protein content of a normal diet. Their elimination from the diet inevitably alter the macro- and micronutrient composition of a GFD [33,52,56,99,106,135].

Rice, corn and potatoes are widely used as natural substitutes of gluten-containing grains but are generally less nutrient dense. Moreover, processed gluten-free products are often of lower quality and poorer nutritional value compared with their gluten-containing equivalents [9,10,11,106]. A number of nutrient-dense grains, including the pseudo-cereals buckwheat, quinoa and amaranth, represent a safe alternative concerning gluten absence while improving the variety and palatability of the GFD, and are a good source of carbohydrates, protein, dietary fiber, vitamins, minerals, and polyunsaturated fatty acids [136,137].

On a macronutrient level, several studies suggest that a GFD is characterized by lower intake of (complex) carbohydrates and fibers, with subsequent higher protein and fat consumption [30,33,52,56,99,108,113,135,138]. The altered macronutrient dietary patterns can have negative consequences. For example, poor dietary fiber intake can increase the risk of other chronic diseases [99,101,113,135,139]. Additionally, a rise in body mass index (BMI) and increased prevalence of metabolic syndrome has been observed in CD patients after initiation of a GFD [140,141]. 

#### Intake of Micronutrients

Gluten-free cereal products generally contain inferior amounts of iron, folate and B vitamins compared to the products they are intended to replace, contributing to a lower intake of these nutrients by CD patients on a GFD [9,10,11]. Adult CD patients on a GFD failed to achieve the recommended daily amount of iron intake in 46%–54% of cases (see Table 2) [52,99,101,104,113,135,138]. In contrast, other studies suggested that iron intake is similar or even higher compared to healthy controls and Ohlund et al. found that merely 8% of pediatric CD patients failed to meet the recommended intake for iron [52,99,101,135,138]. Likewise, the intake of folic acid in adult CD patients is generally below recommendations (in 35%–98% of patients), but was shown to be similar or higher compared to the reference population in most studies [33,49,52,99,101,113,138]. In children with CD, folic acid intake has been shown to meet recommendations, similar to controls [138]. Additionally, the correlation between dietary intake and plasma levels of folate was found to be poor [33]. Vitamin B6 intake has been reported to be below recommendations in 33% of adults, which was significantly higher compared to 17% in controls in a cross sectional case-control study by Valente et al., and in 8% of pediatric patients adhering to a GFD [111,113]. A low nutrient density of the other B vitamins thiamine (B1), riboflavin (B2) and niacin (B3) has been found in the diet of treated pediatric CD patients compared to healthy controls [113].

Surprisingly, the intake of nutrients not present in cereal products appears to be low as well in patients on a GFD. Animal products (meat and dairy) represent the only dietary source of vitamin B12 and are not restricted in a GFD. Therefore, the intake of vitamin B12 in CD patients on a GFD appears to meet recommendations, both in adults and children [33,113,138]. However, Kinsey et al. showed a low mean daily vitamin B12 intake in 10% of patients aged above 65 [99]. The intake of vitamin D has been suggested to be below recommendations in both pediatric patients (68% with a poor intake) and in adult patients (53%–100%) on a GFD, even below the generally low intake in healthy subjects [99,113,138]. 

Although minerals are located in the germ of grains and are not expected to be completely lost during the refining process, there is evidence that suggests low intake of minerals in CD patients on gluten elimination. Due to secondary lactose intolerance, calcium consumption might be low in CD patients. The intake of calcium in CD patients adhering to a GFD failed to achieve recommendations in 12%–78% of adults and in 8%–54% of children, although it has been shown to be similar or above intake-levels in reported reference populations [48,52,99,101,104,107,108,109,110,113]. Low calcium intake has a negative effect on BMD, which can already be impaired at time of CD diagnosis. Patients on a GFD also failed to achieve the recommended daily amount of magnesium intake in 29%–76% of children and in 28%–50% of adults, in line with the general population [48,52,101,113,138]. Furthermore, zinc intake was reported to be below recommendations in 11%–58% of adult CD patients adhering to a GFD, in line with intake in healthy adults in the reference population [52,101,108]. Pseudo-cereals contain higher amounts of minerals compared to other gluten-free cereals such as rice and maize and, therefore, provide a good alternative for CD patients [142].

Nutrient inadequacies in processed gluten-free products and incorrect dietary choices may contribute to inadequate nutrient intake identified in CD patients adhering to a GFD. In line with this, nutrient imbalances were more evident in children with strict compliance to a GFD than non-compliant patients [100]. A different choice of grains has the potential to improve the nutrient profile of the diet for individuals with CD, but attention must be paid to the complete dietary pattern [137]. Taken together, attention is required to the dietary shortcomings of micronutrients in CD patients adhering to a GFD.

## 5. Discussion

The current review of the available literature indicates that both newly diagnosed and CD patients following a GFD frequently exhibit nutrient deficiencies. Second, nutrient deficiencies are associated with short- and long-term manifestations and complications of CD [2,3,4,49]. However, the clinical impact of nutrient deficiencies and their modulation by treatment in CD patients are still unclear based on the available literature. Our current review identifies a high degree of variation between reports and a deficit in scientific evidence supporting a consistent role of nutrient deficiencies in CD on several levels. For example, no high-quality studies were identified that assessed deficiencies of iron, vitamin D, vitamin B12, folic acid, zinc or magnesium status in adult CD patients on a GFD. In general, after the analysis of the level of evidence of the reports reviewed in this work, only low- to moderate-level studies and practically no high-quality, high-level evidence studies were available. As a result, the evidence base is not strong enough to pronounce sound recommendations on CD management concerning nutrient status. Therefore, we suggest evaluating nutrient levels at the time of diagnosis as well as regularly during follow-up, until further research provides the evidence base necessary to generate more detailed recommendations. The results of this review show the importance of a nutritionally balanced dietary pattern as a part of CD management that can still lead to clinical improvement in most patients. Health providers should focus not only on gluten avoidance, but also on a balanced diet with respect to macro- and micronutrients in all CD patients. Early education of patients by a skilled dietitian with expert knowledge in CD is needed to address the achievement of adequate (micro) nutrient intake in patients on a GFD. Naturally gluten-free foods should be recommended as these have a higher nutritional value in terms of protein and fiber provision and vitamin content as opposed to the commercially purified gluten-free products [9,10,11]. Fortification of gluten-free foods needs to be considered and should at least match the micronutrient content of the foods they intend to replace. Targeted supplementation based on laboratory findings or a multivitamin and mineral supplement could be beneficial to the health status and recovery of individual CD patients, although care should be taken to avoid hypervitaminosis. Thus, vitamin B12 as well as folic acid supplementation have been shown to be clinically relevant in preventing or reducing neurological and psychological symptoms in CD patients. In children and adolescents, vitamin D and calcium supplementation are of particular importance to improve BMD. Interestingly, most studies evaluating the role of dietary supplements in CD management mainly focus on the restoration of blood levels as opposed to the effects on clinical consequences of nutrient deficiencies. However, if clinically relevant outcomes are investigated, the focus lies largely on long-term effects of nutrient supplementation on parameters such as BMD. Thus, short-term adverse effects of nutrient deficiencies are not taken into account. IDA and iron deficiency for example, may improve on a GFD regardless of the use of iron supplements, but may improve faster in patients using supplementation. A prolonged duration of recovery could have potentially harmful effects on the patients’ health and/or psychosocial well-being. A prolonged period of IDA in pediatric CD patients could, for instance, result in a longer period of fatigue and weakness, impairing school performance and social life. In adult CD patients, this burden could also have an additional societal impact when resulting symptoms lead to reduced work performance or prolonged sick leave [143,144]. Taking this into account, the time it takes for nutrient deficiencies to restore after initiation of GFD treatment should be of interest in future studies. Moreover, efforts should be made to generate more evidence supporting sound recommendations regarding the effects of treatment and monitoring of CD patients in which a nutrient deficiency or a clinical consequence of deficiency has been detected.

As mentioned above, certain limitations of this review’s methodology should be taken into account. These limitations, combined with several weaknesses in the current literature, especially concerning the assessment of nutrient deficiencies, should be addressed and summarized. This is not only of great importance for interpretation of the presented overview, but also to provide recommendations concerning future research efforts. Although this narrative review has not been constructed as a systematic review, an extensive literature search of three databases has been conducted aiming to give as comprehensive an overview as possible. Nevertheless, this review is not a complete representation of all studies, but rather provides an overview of the current state of knowledge. High-quality studies were also included in this review, however, due to their scarcity some studies of lesser quality were considered in order to provide a general overview (see Supplementary Table S1). Through conducting the literature search and selection by two investigators and broad screening for eligible articles, we aimed to minimize the risk of selection bias, but nonetheless we still present a narrative review.

A lack of consensus could be identified concerning several methodological aspects. This results in high variability in conducted studies concerning patient selection, chosen cut-off points for nutrient values and varying quality of assays measuring nutrient levels. Therefore, we present the reported percentages of patients with micronutrient values below the reference points, as chosen by the researchers of the respective studies. A uniform, clinically relevant cut-off value was thus not selected, as this can be controversial regarding several nutrients, for instance concerning vitamin D concentrations [145,146,147]. Another relevant point of concern is whether micronutrient levels measured in the blood (serum or plasma) are an accurate indicator of the overall status of a certain micronutrient in the entire body. This relates, for example, to zinc and magnesium measurements. It was suggested that other biomarkers in the blood could be a more accurate indicator of zinc status in the body [148]. Similarly, there are conflicting views on the use of magnesium levels in blood or urine as an assessment for magnesium status [149]. Notably, few studies make a distinction in the severity of nutrient deficiencies, but rather only report on the number of patients not meeting the lower cut-off value (see Supplementary Table S1) [40,41,42]. In order to truly evaluate the scope of the problem of nutrient deficiencies in CD, future studies might separately report on patients with sub-optimal nutrient values and patients with severe deficiencies. Overall, these shortcomings reflect general limitations of the current literature on this topic and could be viewed as an important outcome of this review. A consensus on these methodological issues should thus be achieved before studies assessing the subject matter elaborated upon in this review are executed.

## 6. Summary and Conclusions

Both newly diagnosed CD patients and patients adhering to a GFD frequently demonstrate nutrient deficiencies which can have important clinical consequences. Nutrient deficiencies with known clinical relevance in CD patients include iron, vitamin D, folic acid, zinc and calcium at the time of diagnosis and iron, vitamin D, vitamin B6 and zinc during treatment with GFD.

In newly diagnosed CD patients these deficiencies may reflect the loss of absorptive surface area as well as functional capacity. Following the elimination of gluten from the diet, improvement in small intestinal histology occurs gradually, especially in adult patients. Consequently, recovery of nutrient deficiencies after diagnosis takes time. Nevertheless, even during long-term GFD treatment in CD patients with biopsy-proven remission, these patients may still show nutrient deficiencies due to insufficient nutrient intake. 

Several known comorbidities, e.g. osteoporosis, anemia and neurological symptoms, are possible indicators of the impact that impaired nutritional status can have on CD patients. In pediatric CD patients, growth or impaired sexual maturation can be consequences that highlight the potential impact of impaired nutritional status in this patient group. Consequentially, assessment of nutritional status should be a part of both CD diagnosis and during follow-up. Additionally, dietary education for CD patients focusing not only on gluten elimination, but also the need to balance dietary patterns is important and should receive more attention.

Evidence on the benefit of nutrient supplementation on mucosal healing, correction of nutrient deficiencies or recovery from comorbidities associated with CD is inconclusive. While vitamin B12 and folic acid supplementation appear beneficial, no evidence was found supporting favorable effects of calcium and vitamin D supplementation on decreased BMD in CD. 

This review, although providing evidence for the relevance of nutrient status in CD at diagnosis and during treatment with a GFD, shows a noteworthy lack of high-quality evidence and a high degree of variability in the current literature regarding several relevant aspects. This concerns especially the lack of research in pediatric CD patients on a GFD and the consequences of impaired nutritional status on child development. The latter undoubtedly represents an important aspect in clinical care of these pediatric CD patients. 

The causes of an impaired nutrient status in CD are currently only poorly understood. As improvement of nutritional status is of great importance for patients, further studies are warranted with comprehensive assessment of nutrient status in untreated and, even more importantly, treated CD patients are warranted. Studies should differentiate between adult and pediatric study populations and evaluate the need for optimal timing and dosing of supplementation as part of CD management. Prospective cohort studies would be expedient to further investigate the prevalence of certain nutrient deficiencies, their resulting health complications, and the potential role of nutrient supplements. It is of importance that future cohort studies are conducted on a multinational scale. This would not only account for differences between patient groups with different dietary habits, but also for patient groups that receive different types of medical care, from general practitioners and primary care facilities to specialized tertiary centers. Furthermore, researchers should pay more attention to the methodological aspects of assessing nutrient deficiencies, specifically regarding the type of measurement and appropriate reference values, clearly distinguishing between patients with sub-optimal nutrient levels and those with severe deficiencies. This will enable a more accurate evaluation of the scope of the problem and the clinical efficacy of treatment in the future. Long-term prospective studies could also provide evidence on the time it takes for nutritional deficiencies to recover and the factors influencing this process. This may shed light on the question whether we should strive to achieve a more rapid recovery of nutrient levels in CD patients after diagnosis. It could further clarify the matter if this can be achieved through attention to diet alone, or through addition of nutrient supplementation. In order to conduct valuable research addressing nutrient status in CD, it is crucial to reach a consensus on cut-off values of nutrient levels as well as the adequate techniques that should be used to assess them. When investigating the causes for the occurrence of certain deficiencies, the addition of a group of subjects (controls) following a GFD that do not have CD could be of additional value. This is currently more feasible, as GFD is becoming increasingly popular in healthy individuals without CD or in patients with non-celiac gluten sensitivity. This will yield important further knowledge to improve overall management of CD. The goal of this should be to relieve symptoms, recover the intestinal mucosa, and reverse the consequences of CD-related malabsorption, while enabling patients to secure a nutritionally adequate GFD.

## Figures and Tables

**Table 1:** Overview of nutrient deficiencies at diagnosis and during follow-up on a gluten-free diet.

Nutrient	Percentage of Untreated CD Patients with Circulating Levels below Reference Value (%)	Percentage of Treated CD Patients with Circulating Levels below Reference Value (%) Short Term Follow-up, <2 years on a GFD (Time on GFD)	Percentage of Treated CD Patients with Circulating Levels below Reference Value (%) Long Term Follow-up, >2 years on a GFD (Time on GFD)	Percentage of Individuals in the General Reference Population with Circulating Levels below Reference Value (%)
**Adults**				
Iron (iron/ferritin)	6%–82% [15,16,17,18,19,20,21,22,23]	Serum iron: 44% Serum ferritin: 15% (1 year) [24] *	data not available	17 % [23]
Vitamin D (25(OH)D)	5%–88% [18,20,25,26,27,28]	50% (1 year) [29] *	7.6% (5 years) 0% (mean 4 years for men, 9 years for women) [29,30] *	50% [25]
Calcium	0%–26% [17,19,20,28,31]	0% (1–2 years) [17]	data not available	data not available
Vitamin B12	5%–19% [16,17,18,20,22,32]	data not available	0% (8–12 years) [33]	7%–17% [18,23]
Vitamin B6 (vitamin B6/Plasma pyridoxal 5 phosphatase)	15% [18]	data not available	37% (8–12 years) [33]	0% [18]
Folic acid (folic acid/folate)	11%–75% [15,16,17,18,19,20,22,23,31,32]	data not available	20% (8–40 years) [33] *	4%–14% [18,23,32]
Zinc	67% [18]	30%; (1 year) [24] *	20% (range 8 months–7 years) [34] *	data not available
Magnesium	13%–17% [35,36] *	data not available	data not available	data not available
**Children**				
Iron (iron/ferritin)	12%–82% [22,23,37,38,39]	Serum iron: 5%–10% Serum ferritin: 21%–27% (6 months–2 years) [38,39]	Serum iron: 4%–8% (3–5.5 years) [38]	17% [23]
Vitamin D	0%–70 % [25,38,39,40,41,42]	0%–57% (6 months–2 years) [38,39,40,41]	12%–25% (2–5.5 years) [38]	4%–30% [25,40,42]
Calcium	0%–41% [37,38,40,43,44,45,46]	0% (6 months–2 years) [38,40]	0% (3 years–5.5 years) [38]	0% [40]
Vitamin B12	1%–14% [22,23,37,38]	0%–1% (6 months–2 years) [38,39]	0% (3–5.5 years) [38]	7% [23]
Vitamin B6	data not available	data not available	data not available	data not available
Folic acid	14%–31% [22,23,37,38]	0%–3% (1–2 years) [38]	0% (3–5.5 years) [38]	14% [23]
Zinc	19%–72% [37,39,47]	16%–18% (6–18 months) [39]	data not available	data not available
Magnesium	7%–11% [40,48]	data not available	4% (11 years; range 3–17) [48]	0% [40]

Overview of reported percentages of adult and pediatric CD patients with a nutrient deficiency at the moment of diagnosis and during follow-up with a GFD. All reported values are summarized in ranges, with the corresponding studies referenced in square brackets. The duration of GFD is mentioned in brackets in columns three and four. * Studies that did not meet quality criteria were only included if no other eligible article existed for that nutrient level and are marked by an asterisk. Abbreviations: celiac disease (CD); gluten-free diet (GFD).

**Table 2:** Overview of dietary intake of nutrients on a gluten-free diet in celiac disease patients and reference populations.

Nutrient	Percentage of CD Patients with Nutrient Intake below Recommendations (%)	Percentage of Individuals in the General Reference Population with Nutrient Intake below Recommendations (%)
**Adults**		
Iron	46%–54% [52,99,101,104,107]	14% [52,99]
Vitamin D	53%–100% [99,107,108]	data not available
Calcium	12%–78% [99,101,104,107,108,109,110]	6%–29% [52,99]
Vitamin B12	10%–61% [99,107,111]	1%–65% [99,111]
Vitamin B6	33% [111]	17% [111]
Folic acid	35%–100% [54,101,107,110,111,112]	3%–100% [52,99,111]
Zinc	11%–58% [52,101,107,108,110]	30% [52]
Magnesium	28%–50% [52,101]	29% [52]
**Children**		
Iron	8% [113]	43%–79% [100,110]*
Vitamin D	68% [113]	data not available
Calcium	8%–54% [48,113]	86% [110]*
Vitamin B12	0% [113]	data not available
Vitamin B6	8% [113]	data not available
Folic acid	80% [110]	57% [110] *
Zinc	40% [110]	43% [110] *
Magnesium	29%–76% [48,113]	data not available

Overview of reported percentages of adults and children with CD and of general reference populations with nutrient intake below the recommended levels. All reported values are summarized in ranges, with the corresponding studies referenced in square brackets. * Studies that did not meet the quality criteria were only included if no other eligible article existed for the intake of that nutrient and are marked by an asterisk. Abbreviations: celiac disease (CD).

## Supplementary Materials

The following are available online at https://www.mdpi.com/2072-6643/12/2/500/s1: Table S1: Overview of studies included in Table 1 and Table 2, describing nutrient deficiencies in celiac disease patients with active and treated CD or nutrient intake in CD patients on a gluten-free diet.
