# Supplementary material for: Narrative Review: Nutrient Deficiencies in Adults and Children with Treated and Untreated Celiac Disease
Source: Nutrients. 2020 Feb 15;12(2):500. doi: 10.3390/nu12020500 (PMC7071237; doi:10.3390/nu12020500)
Supplement: Supplementary file 1 [file nutrients-12-00500-s001.pdf]

Supplementary Material:

Table S1. Overview of studies included in Tables 1 and 2, describing nutrient deficiencies in celiac disease patients with active and treated CD or nutrient intake in CD patients on a gluten-free diet.

Paper	Study design	Subjects	Number of subjects	Age and demographics	% female	Outcome parameters	Description of tests and reference values used for biochemistry	Level of evidence (OCEBM 2009)
Tikkakoski *et al.* 2007	Prospective cohort study	Patients screened for CD	36 CD patients from 1900 adults screened for CD	Adults (18-64 years); in Finland	73%	Serum iron (median and number of patients with values below reference range of 9- 34 umol/l	Test not described Reference ranges: 9-34 umol/l for S-Fe, 4.5-34 nmol/l for folate	2b
Harper *et al.* 2007	Retrospective chart review	Adult CD patients Reference cohort: NHANES III	400	Mean age at diagnosis 46.5 years (SD 16.2); USA	67%	Hematologic blood values and biochemistry, Marsh grade	Not specified	2b
Vilppula *et al.* 2011	Prospective cohort study	Elderly population from the GOAL cohort	New CD patients: 35, screen detected from 2815 subjects	Median age at diagnosis: 61 (range 52-76); Finland	57%	Celiac serology and HLA typing, clinical symptoms, BMD, fracture history, BMI, hematologic values and biochemistry	Not specified	3b
Wierdsma *et al.* 2013	Cross-sectional single center cohort study 2005–2012	Consecutively diagnosed adult CD patients and healthy controls	CD: 80 Healthy controls: 24	(aged 18–75 years); Netherlands	65%	Marsh grade, BMI, biochemistry	Test not described, reference values provided: Vitamin A [1.2–3.0 nmol/L]; Vitamin B6 [13–80 nmol/L]; Folic acid [>5.6 nmol/L]; Vitamin B12 [150–700 pmol/L); Zinc [11–19 nmol/L]; Vitamin (25-hydroxy) D [30–150 nmol/L]; Hemoglobin [M 8.5–11/F 7.5–10 mmol/L]; Ferritin [20–250 µG/L]	3b
Zanini *et al.* 2013	Retrospective single center cohort study 1990-2010	Consecutively diagnosed CD patients	1408	Adult CD patients; Italy	73%	Demographics, anthropometrics, clinical manifestations, biochemistry, hematology, celiac serology, BMD	Not specified	2b
Schosler *et al.* 2016	Retrospective multicenter chart review 2008-2013	CD patients	93	>15 years ; one county in Denmark	76%	GI symptoms, anthropometrics, biochemistry, and BMD	Test not described; Definition of abnormalities: Anemia: hemoglobin <57.3 mmol/l (women) and <58.3 mmol/l (men); Folate deficiencies: folate < 9 nmol/l; PTH elevation: parathyrin >6.9 pmol/l; vitamin B12 deficiency: cobalamin <200 pmol/l; iron deficiency: ferritin < 22 mg/l; vitamin D deficiency: 25-hydroxy-vitamin D2+D3 < 50 nmol/l; elevated alkaline phosphatase: alkaline phosphatase > 105mmol/l; low ionized calcium: < 1.18mmol/l.	2b

Sansotta *et al.* 2018	Single center retrospective chart review 2002-2015	Pediatric and adult CD patients	Children: 227 Adults: 327	Median (range) Children: 8.5 years (1.28–17.89) Adults: 38.9 years(18.3–75.7); USA	Children: 66% Adults: 78%	GI and EI symptoms, prevalence of IDA	Not specified	2b
Berry *et al.* 2018	Single center prospective observational study 2012-2013	Consecutive patients, adult CD	103	Age at presentation (baseline) 26.15 ± 13.3 years; North India	53%	Clinical presentation, hematology, presence of anemia and folate, vitamin B12 and iron deficiency	Anemia: hemoglobin < 12 g/dL (females) and <13 g/dL (males) (WHO criteria); Iron deficiency: serum ferritin <30 ng/mL, serum iron <30 µg/dL, total iron binding capacity > 400 µg/dL, and percentage saturation < 15%; Folate deficiency: serum folate <4.0 ng/mL; vitamin B12 deficiency: serum B12 levels <200 pg/mL. Serum folate and B12 levels measured using a competitive immunoassay	1b
Haapalahti *et al.* 2005	Retrospective cohort study	CD (clinical diagnosis and through screening) and controls from cohort of healthy school children studying risk factors for type 1 Diabetes	CD patients: 26 Healthy controls: 29	16 to 25 years; Finland	69%	Biochemistry, celiac serology, BMI	Used reference values of Oulu University Hospital; serum ferritin <25 mg/L in females and <20 mg/L in males, whole blood folate <85 nmol/L, serum pre-albumin <0.23 g/L in females and <0.24 g/L in males and serum vitamin B12-vitamin <180 pmol/L were considered as low concentrations.	2b
*Kemppainen *et al.* 1998	Single center, Prospective intervention study on use of oats in CD 1988-1990	Adult CD patients, newly diagnosed	New CD: 40	Male:47 ± 12 (24–65) Female ± 13 (18–62); Finland	70%	Marsh, energy intake, reported symptoms, food records, anthropometrics and biochemistry	Methods measurement described for: Ferritin: immunoluminometric assay; Vitamin B12: radioisotope dilution assay; Zinc: atomic-absorption spectrophotometry literature references for assays provided for vitamin D, E and A no reference values provided	2b
Lerner *et al.* 2011	Multi center cross sectional case control study	5 groups: New Pediatric and adult CD patients, Pediatric patients with nonspecific abdominal pain and their parents (adult control group)	Pediatric CD: 110 Adult CD: 22 Pediatric controls: 56 Adult controls: 84	Pediatric CD: 6±4 years (Israel) and 4±4 years (Spain) Pediatric controls 8±5 years Adult controls: 39±8 years (Israel) and 44±13 years (Spain); Israel and Spain	Children: 47 % Adult: 35%	Serum vitamin D Symptoms, familial disease, ethnicity, CD serology and biochemistry, histology	Measurement 25(OH) vitamin D serum concentration: commercial kit, LIAISON® 25(OH)D Assay (DiaSorin, Italy); Deficiency defined as <20 ng/ml according to Saintonge et al (2009)	3b
Chakravarthi *et al.* 2012	Cross sectional cohort study 2008-2009	Adult new CD	54	Mean ± SD age of 30.6 ± 9.3 years (range 18–50);	56 %	BMD, biochemistry, CD serology, Marsh classification	25 (OH) vitamin D levels were estimated by radioimmunoassay (normal values are >20 ng/mL).	3b

				India				
Garcia-Manzanares *et al.* 2012	Single center cross sectional study	Adult-onset consecutive CD patients	40	18 - 68 (mean 44.25 years); Spain	90%	CD serology, HLA type, Marsh classification, biochemistry and urine, WHO fracture risk assessed by FRAX tool, BMD	Not specified	3b
Posthumus *et al.* 2017	Retrospective single center cross-sectional study	Adult-onset CD	283	Mean age at diagnosis 39 ± 12 (range: 18–67) years; Netherlands	78%	Biochemistry, Marsh classification, BMD	Not specified	2b
*Kemppainen *et al.* 1999	Prospective study 5 year follow-up 1990-1991	Adult new CD	28	Age (years) Female: 44.1± 13.6 (23.0–66.0) Male: 48.6±12.3 (24.0–65.0); Finland	68%	BMI, BMD, medication and supplement use, four day food record, calcium, vitamin D and PTH	Serum 25-hydroxy vitamin D assay described by Törnquist and Lamberg-Allardt (1987) At 5 year follow-up: serum 25(OH)D concentration measured by commercial radio immunoassay (Incstar, Stillwater, MN) (blood drawn in autumn)	3b
*McFarlane *et al.* 1995	Single center cross sectional cohort 1991-1992	Adult-onset CD	55	Men: 50.2 years (range 27.0-65.0 years) Women: 51-3 years (range 33.6-69.1 years); United Kingdom	82%	Dietary assessment, BMI, biochemistry, BMD	Not specified	3b
Fernandez *et al.* 2010	Retrospective chart review 1990-2008	Adult-onset CD	68	Median age 36 years (range: 18-65); Spain	74%	Biochemistry, homocysteine levels, C677T mutation in methylene-tetrahydrofolate reductase (MTHFR) gene	Not specified	3b
*Hallert *et al.* 2002	Prospective multi-center cohort study 23% loss to follow up; compared to Nordic reference population and historic reference population	Adult CD 8–12 years of treatment with GFD; biopsy proven remission	30; Nordic reference population: 592 Historic reference population:504	Mean age 55 years; 95% confidence interval (CI), 53.2–56.8; Sweden	60%	Biochemistry, plasma homocysteine, CD serology, 4-day food record, anthropometrics, Marsh classification, basal metabolic rate predicted using Schofield equations	Vitamin B-12: radioimmunoassay; plasma pyridoxal 5′-phosphate & total plasma homocysteine: enzymatic photometry with high-performance liquid chromatography separation (Mimelab AB, Söråker, Sweden) Reference range: Blood hemoglobin (g ⁄L): 130–165 for males, 120–150 for females; Plasma ferritin (lg ⁄L): 30–230 for males, 30–150 for females; Serum calcium (mmol ⁄L): 2.2–2.6; Serum zinc (lmol ⁄L): 11–17.7	2b
*Crofton *et al.* 1983	Intervention study evaluating zinc absorption	Adult CD, newly diagnosed and on a GFD; Healthy controls	CD: 22 Controls: 15	CD: Untreated: 47 (range 16-78) Treated: 38 (range 23-57)	CD: 68% Controls: 20%	Plasma zinc concentrations, zinc tolerance test	Not specified	4

				35 (range 25-50);<br><br>UK			
*Boyd *et al.* 1985	Single center retrospective cohort study 1969-1983	Adult and Pediatric consecutive CD patients	50	Range 13 to 71 years (median, 30 years);<br><br>UK	68%	Presenting symptoms, anthropometrics, CD serology, test for malabsorption	Hemoglobin <12 g/dl; Folic acid <2.0 pg/l; Vitamin B12 <200 ng/l; Iron- men <16 pmol/l, women <11 pmol/l; calcium < 2.20 mmol/l; magnesium <0.70 mmol/l; zinc <8.4 pmol/l	4
Kuloglu *et al.* 2009	Retrospective single center chart review 1998-2006	Pediatric CD	109	Mean age 8.81 ± 4.63 years;<br><br>Turkey	60%	Biochemistry, CD serology and autoantibodies, BMD, HLA genotype, BMI, anthropometrics,	Not specified	3b
Wessels *et al.* 2016	Single center retrospective chart review;<br>2009-2014	Pediatric CD, newly diagnosed and on routine check up	182	Mean age at diagnosis 6.3 years (±4.3) ;<br><br>Netherlands	65%	Biochemistry, Marsh grade, HLA genotype, CD serology	Ferritin, folate, vitamin B12: electrochemiluminescence immunoassay using Roche Modular E170; Roche Diagnostics, Basel, Switzerland); Calcium: Roche Modular P800; Roche Diagnostics); vitamin D: electrochemiluminescence immunoassay using Roche Modular E170; Roche Diagnostics)<br><br>Reference values: Hemoglobin: age <7 y <6.9 mmol/L (<11.0 g/dL), age 7-15 y <6.5 mmol/L (<10.4 g/dL), age >15 y <6 mmol/L (<9.6 g/dL); Ferritin: age <5 y <12 ug/L , age ≥5 y <15 ug/L;<br>Folate: <10 nmol/L (<4.45 ng/mL); Vitamin B12 <150 pmol/L (203 pg/mL); Calcium<2.15 mmol/L;<br>Vitamin D (25[OH]D) <50 nmol/L (<20.8 ng/mL)	2b
Deora *et al.* 2017	Single-center cohort study; at diagnosis, 6 and 18 months after starting a GFD, including patients receiving supplementation 2012-2016	Consecutive pediatric CD patients (<17 years)	140	Mean age 7.8±4.01 years;<br><br>Canada	62%	Biochemistry, CD serology, marsh grade, dietetic assessment of GFD	Not specified	4
Zanchi *et al.* 2008	Prospective case-control study<br>2004-2005	Untreated pediatric CD patients and healthy children	CD: 54<br>Controls: 60	CD: mean age: 7.4 ± 4 years; range, 1.5 to 15 years<br>Controls: mean age: 8 ± 3 years; range, 2 to 16 years;<br><br>Italy	CD:59%<br>Controls: 58%	Biochemistry, CD serology, BMD, marsh grade	Calcium (normal values, 9.2 to 11 mg/dL), magnesium (1.80 to 2.30 mg/dL), 25(OH) vitamin D3 (20 to 120 ng/mL),	1b
Mager *et al.* 2012	Single center prospective cohort	Pediatric CD patients	43	At diagnosis 9.4±4.2 years (3 to 17);	64.8%	BMD, marsh grade, anthropometrics, vitamin D/K status, diet, physical activity and sunlight exposure	Performed in the Core Laboratory at the UA Hospital according to standard methodologies;<br>serum 25(OH)-vitamin D:	2b

	Diagnosis and 1 year follow up (23% loss to follow-up)<br>2009-2010			Canada			deficient <50 nmol/l,<br>suboptimal 50–75 nmol/l, sufficient >75 nmol/l according to Holick et al., 2007;<br>Calcium (mmol/l) 2.1–2.6; Magnesium (mmol/l) 0.7–1.0 mmol/l	
Tokgöz *et al.*<br>2018	Single center case control<br>2015-2016	Consecutive Pediatric CD patients; healthy controls	CD: 52<br>Controls: 50	Range 0-18 years<br>Median age CD: 9 ± 4.3 years for CD;<br>Median age controls:<br>8.7 ± 5.2 years;<br><br>Turkey	CD: 52%<br>Controls:50%	Vitamin D, K, E,A, CD serology, Marsh grade, anthropometrics, symptoms at diagnosis	Vitamin D: Chemiluminescence method in Architect hormone autoanalyzer (C8000 Architect, Abbott, Abbott Park, IL, USA).<br><br>vitamin D insufficiency < 30 ng/ml, vitamin D deficiency < 20 ng/ml	2b
*Kavak *et al.*<br>2003	Case control study<br>2000-2001	Pediatric CD patients new and on a GFD; gender- and age matched healthy control subjects;<br>None received supplements	Untreated CD: 34<br>CD on 1 year GFD: 28<br>Matched controls: 64	New CD: mean age 7.6 ± 4.7 years (range, 2–15 years);<br>CD on GFD: mean 7.9 ± 4.7 years (range, 2–16 years);<br><br>Turkey	New CD: 62%<br>CD on GFD: 57%<br>Controls: 47%	Biochemistry, BMD	Serum calcium: normal, 8.8–10.8 mg/dL; 25 -hydroxy vitamin D (25-OHD3): normal 9–45 ng/mL, determined with radioimmunoassay (RIA kit, Diasorin Inc., Stillwater, MN), intra-assay coefficient 6.1%,inter-assay coefficient 7.1%	4
Tau *et al.*<br>2006	Longitudinal and prospective study<br>1995-2001	Pediatric CD consecutive patients received 1000 IU vitamin D per day during first 3 months of GFD;<br><br>Compared to historic cohort of French children (Glastre et al., 1990)	24	Mean age at diagnosis:<br>4.9±74.30 years (range 1–11.7 years);<br><br>Argentina	75%	BMD, biochemistry	Serum calcium atomic absorption spectrophotometry (normal value: 2.20–2.70 mmol/l); 25 hydroxy vitamin D in-house radiocompetitive protein-binding assay (Delvin et al., 1980) normal values: 92.5722.5 nmol/l in summer and 62.57 25 nmol/l in winter	3b
Volkan *et al.*<br>2018	Prospective case-control study<br>2015-2016	Pediatric CD;<br><br>Age- and sex-matched healthy controls	New CD: 26<br>CD on GFD: 46<br>Controls: 30	Mean age<br>CD: 11.69±3.04<br>Controls: 12.27±2.12;<br><br>Turkey	Not specified	BMD, vitamin D, K, CD serology	Not specified	3b
Rawal *et al.*<br>2010	Prospective RCT<br>2006-2007	Consecutive Pediatric CD patients with and without zinc suplpementation	134	Mean age 6.71± 3.45 years (range 2–14 years);<br><br>India	38%	Biochemistry, hematology, CD serology	Plasma zinc measurement: atomic absorption spectrophotometer (PERKLIN ELMER 400)<br>Normal values (standardized in lab): 70–110 mgdl^{-1}	1b
Rujner *et al.*<br>2004	Single center cohort study	Pediatric CD, new and on GFD<br>Healthy controls	New CD: 28<br>On GFD: 41<br>Controls: 8	Female: mean age 13.04 years, range 5.92–18.3	CD: 38%	Presenting symptoms, Mg retention, serum, erythrocyte, and urine Mg	Tissue magnesium deficiency: magnesium retention > 40% of intravenous load;<br>Mg concentrations in serum, erythrocyte, and urine: flame atomic absorption spectrometry;	2b

				Male: mean age 12.3 years, range 5.9–16.7;<br><br>Poland		concentration, Mg dietary intake	Normal values Mg concentrations:<br>Mg serum (mmol/l)<br>(min–max) 0.7–1.04m, Mg erythrocyte (mmol/l) 1.8–2.4, Mg in urinary excretion (mmol/24 h/1.73m^2) >2.0	
Wild *et al.*<br>2010	Prospective single-center cohort study<br>2007-2008	Consecutive CD strict GFD for 6 months or more;<br>2 control populations:<br>National Diet and Nutrition Survey of Adults (NDNS) Northern (n = 256)<br>UK Women's Cohort Study (UKWCS) (n = 708)	93	Median age (years)<br>Male: 63 (18–74)<br>Female: 53 (21–79);<br><br>UK	67%	Nutrient intake (prospective validated 5-day food diary), CD serology, comorbidities, reported dietary supplementation	EPIC validated food diary (Bingham et al. 2001)	2b
Kinsey *et al.*<br>2008	Cross-sectional postal survey,<br>CD diagnosis confirmed by gastroenterologist	Adult CD<br>Reference population:<br>NDNS Henderson et al., 2002, 2003	49	Mean age 58.6 (±17) years;<br><br>UK	76%	Three-day food diary, food questionnaire; Calculated mean daily intake of macro and micronutrients	Not specified	3b
Shepherd *et al.*<br>2013	Prospective cohort study	New CD and, CD on a GFD	New CD: 50<br>CD on GFD: 55	Median age 44 (range 18–71) years;<br><br>Australia	76%	Hematology, biochemistry, Marsh grade, seven-day prospective food record, structured interview: symptoms, demographics, anthropometry, previous dietary patterns, medical details	Detailed protocol, Measuring cups, spoons and reference diagrams provided	2b
Thompson *et al.*<br>2005	Cross sectional survey study	Adult CD patients	47	Mean age 51 years (SD:11) range 21-73 years;<br><br>USA	83%	Three-day estimated self-reported food records	Not applicable	4
Mijatov *et al.*<br>2016	Prospective cohort study	Women with CD on strict GFD	40	Mean age in years 66.4 (range 41-109);<br><br>Slovenia	100%	Three-day food diary; Resting metabolic rate, physical activity level	Not applicable	3b
*Grehn *et al.*<br>2001	Cross sectional cohort study<br>1996-1997	Adult CD patients on GFD<br>Reference population:<br>Swedish national dietary survey in 1989, Becker et al.	49	45-64 years;<br><br>Sweden	65%	4-day food record, biochemistry	Blood samples analyzed according to local routines, except for zinc: atomic-absorption spectrophotometry	3b
*Collins *et al.*<br>1986	Single center cross sectional cohort	Adult CD patients	18	Mean age 44 years (range 18-59);<br><br>UK	50%	Anthropometrics, symptoms, dietary history assessed by dietician	Not applicable	4

Pham-Short *et al.* 2017	Case-control study	Pediatric CD and Type I diabetes	T1D+CD: 10<br>Type I diabetes only: 7	Children<br>Type I diabetes+ CD: 14.3 ± 3.6<br>Type I diabetes only: 14.7 ± 2.8;<br><br>Australia	-	Continuous glucose monitoring; Standardized weighed food diary for three days	Food diaries analyzed using Foodworks 7 (Xyris, Australia), which uses Food Standards Australia New Zealand (FSANZ) published AusNut and NUTTAB 2010 databases	3b
Valente *et al.* 2015	Cross-sectional single-blind study<br>2011-2012	CD	CD: 20<br>Healthy controls: 39	Adults 36.3 ± 13.7 years;<br><br>Brazil	65 %	3-day food records, serum concentrations of homocysteine, vitamin B6, B12, and folate determined after overnight fasting	Homocysteine (Immulite2000, Siemens, USA), vitamin B12, and serum folate (Modular e170, Roche, Switzerland) by chemiluminescence, vitamin B6 determined through analysis of PLP, isomer of highest concentration in human plasma, through High-Performance Liquid Chromatography with fluorescence detection Kimura et al 1996, Deitrick et al. 2001	3b
*Bottaro *et al.* 1999	Multicenter (42 participating centers) retrospective cohort study<br>1990-1994	Adult and Pediatric consecutive patients with silent/subclinical CD	1026<br>(644 children, 382 adults)	Mean age:<br>Children: 7.7 ± 4.2<br>Adults: 24.4 ± 12.5;<br><br>Italy	Children: 77%<br>Adults: 71%	Medical history, comorbidities, demographics, symptoms, hematology, CD serology	Not specified	3b
Öhlund *et al.* 2010	Baseline values of a prospective probiotics intervention study 2004	Pediatric CD patients on GFD	25	4-17 years;<br><br>Sweden	72%	5-day food records, anthropometrics,	Use of household measures for quantities; Individual energy requirement calculated according to Nordic Nutrition Recommendations 2004 (NNR-04)	3b

Overview of studies included in Tables 1 and 2 of the review reporting on nutrient deficiencies and dietary nutrient intake at diagnosis and on a gluten-free diet. * Studies that did not meet the quality criteria were only included if no other eligible article existed for the nutrient and are marked by an asterisk. Evidence was assessed using the 2009 Oxford Centre for Evidence-Based Medicine Levels of evidence, were level of evidence could be graded down based on quality and if the study did not match the research question of this review.

Abbreviations: celiac disease (CD), gluten-free diet (GFD), Oxford Centre for Evidence-Based Medicine (OCEBM), serum iron (S-Fe), National Health and Nutrition Examination Survey(NHANES), United States of America (USA), Good Ageing in the Lahti region (GOAL cohort), human leukocyte antigen (HLA), bone mineral density (BMD), body mass index (BMI), Gastrointestinal symptoms (GI symptoms), parathyroid hormone (PTH), Extra intestinal symptoms (EI symptoms), iron deficiency anemia (IDA), world health organization (WHO), WHO fracture risk assessment tool (FRAX tool), United Kingdom (UK), Magnesium (Mg), National Diet and Nutrition Survey of Adults (NDNS) , UK Women's Cohort Study (UKWCS), European Prospective Investigation of Cancer study (EPIC study), C-reactive protein (CRP), Nordic Nutrition Recommendations (NNR).
